# Benralizumab Efficacy in Late Non-Responders to Mepolizumab and Variables Associated with Occurrence of Switching: A Real-Word Perspective

**DOI:** 10.3390/jcm12051836

**Published:** 2023-02-24

**Authors:** Marco Caminati, Alessandro Marcon, Gabriella Guarnieri, Jessica Miotti, Diego Bagnasco, Giovanna Elisiana Carpagnano, Girolamo Pelaia, Rachele Vaia, Matteo Maule, Andrea Vianello, Gianenrico Senna

**Affiliations:** 1 Department of Medicine, University of Verona, 37134 Verona, Italy; 2 Unit of Epidemiology and Medical Statistics, Department of Diagnostics and Public Health, University of Verona, 37134 Verona, Italy; 3 Department of Cardiac Thoracic Vascular Sciences and Public Health, University of Padova, 35129 Padua, Italy; 4 Allergy and Respiratory Diseases, Department of Internal Medicine (DIMI), University of Genoa, 16132 Genoa, Italy; 5 Division of Respiratory Diseases, Department of Medical and Surgical Sciences, Respiratory and Critical Care Unit, University of Bari, Polyclinic University Hospital, 70124 Bari, Italy; 6 Department of Health Sciences, University “Magna Græcia” of Catanzaro, 88100 Catanzaro, Italy; 7 Allergy Unit and Asthma Center, Verona University Hospital, 37134 Verona, Italy

**Keywords:** severe eosinophilic asthma, benralizumab, mepolizumab, switch, clinical response

## Abstract

Overlapping eligibility to different biologics for severe asthma is still challenging, especially when addressing the same target. We aimed to characterize severe eosinophilic asthma patients according to their maintained or reduced response to mepolizumab over time and to explore baseline variables significantly associated with the occurrence of switching to benralizumab. We performed a multicentre retrospective observational study evaluating OCS reduction, exacerbation rate, lung function, exhaled nitric oxide levels (FeNO), Asthma control test (ACT), and blood eosinophil concentrations at baseline and before and after switching occurrence among 43 female and 25 male patients with severe asthma aged 23 to 84 years. Younger age, higher OCS daily dose and lower blood eosinophils at baseline were associated with a significantly higher risk (odds) for switching occurrence. All the patients showed an optimal response to mepolizumab, up to six months. The need for switching, according to the above-mentioned criterion, occurred for 30 out of 68 patients after a median time of 21 months (Q1–Q3: 12–24) from mepolizumab initiation. At the follow-up time-point after the switch (median time: 31 months, Q–Q3: 22–35), all the outcomes substantially improved and no cases of poor clinical response to benralizumab were detected. Although the small sample size and the retrospective design represent major limitations, to our knowledge, our study provides the first real-word focus on clinical variables potentially predicting a better response to anti IL-5r in patients fully eligible for both mepolizumab and benralizumab and suggests that in late non responder patients to mepolizumab, more robustly targeting the IL-5 axis may be effective.

## 1. Introduction

Overlapping eligibility to different biologics for severe asthma is still challenging, especially when addressing the same target. Such is the case of mepolizumab and benralizumab, targeting IL5 and IL5 receptor, respectively [1]. Mepolizumab is a humanized murine IgG1k monoclonal antibody, approved for severe eosinophilic asthma at the dose of 100 mg, administered subcutaneously every 4 weeks [1,2]. Benralizumab is a fully humanized afucosylated IgG1k monoclonal antibody, selectively binding the alfa-receptor for IL-5; it also activates NK cells due to the affinity to human Fcg receptor IIIa (FcgRIIIa) expressed on their surface, enhanced by afucosylation [1,3]. It is approved for severe esoinophilic asthma patients at the dose of 30 mg every 4 weeks for the first three doses, and then every 8 weeks [3].

In fact, the prescription requirements established by the regulatory agencies are extremely similar, not supporting the identification of the best responder to each drug [2,3]. Available markers predicting the clinical response to mepolizumab or benralizumab are not specific or still not applicable in everyday practice [1]. Furthermore, data from real-life settings are few, and do not specifically focus on potential predictors of response [4,5,6].

We aimed to characterize severe asthma patients according to their maintained or reduced response to mepolizumab over time and to explore baseline variables significantly associated with the occurrence of switching to benralizumab.

## 2. Materials and Methods

We performed a retrospective observational study involving five Referral Centres for severe asthma across Italy (Verona, Padua, Genoa, Foggia, Catanzaro). All consenting patients affected by severe eosinophilic asthma and prescribed with mepolizumab during the period January–December 2015 were enrolled (*n* = 68). Severe asthma diagnosis was established according to the ERS/ATS guidelines [7]. In line with that statement, patients requiring high dose inhaled corticosteroids plus a second controller and/or systemic corticosteroids to maintain their asthma controlled, or who were experiencing uncontrolled asthma despite that treatment, were considered as severe asthma patients. Eosinophilic asthma was defined in the presence of 300-blood eosinophils/µL or higher detected at least once in the previous year and 150 cells/µL in oral steroid wash out when the patient was firstly assessed [2,3].

At the time of enrolment, no monoclonal antibodies targeting IL-5 cascade, other than mepolizumab, were marketed in Italy. Failing to maintain a ≥50% reduction of OCS daily dose and ≥50% decrease of exacerbation rate with respect to baseline represented the shared criterion for switching. Unadjusted associations between baseline characteristics and risk of switching from mepolizumab to benralizumab were estimated using Odds Ratios (OR) with 95% CI. Associations were also estimated after adjustment for OCS dose at baseline as a proxy indicator of disease severity.

Changes over time of lung function, exhaled nitric oxide levels (FeNO), Asthma control test (ACT) and blood eosinophil concentrations were also assessed. This secondary analysis was restricted to the subsample of patients with available information, which were 38 and 15 in the non-switch and the switch group, respectively. The 15 patients excluded were not lost to follow up, but the full set of clinical data could not be provided by all the collaborating centres for the time-points after month 6. Baseline and six months follow-up data for both subgroups were analysed. Two further time-points were considered for patients subsequently addressed to switching: the benralizumab-related baseline and the last available follow-up visit. Comparisons of outcomes between time-points were conducted using the Wilcoxon matched-pairs signed-rank test (with the 6-month visit as reference). Missing data were deleted listwise. Statistical analyses were done using STATA release 17.0 (StataCorp, College Station, TX, USA).

## 3. Results

We included 43 female and 25 male patients with severe asthma aged 23 to 84 years. Table 1 summarizes the main characteristics of the study population, including the “non-switch” group maintaining a satisfactory response to mepolizumab and the “switch” group subsequently treated with benralizumab.

Younger age, higher OCS daily dose and lower blood eosinophils at baseline were associated with a significantly higher risk (odds) for switching in univariate analysis. The results were very similar when adjusting for OCS dose at baseline (Table 1), a proxy indicator of disease severity, suggesting that the “case mix” in this study is not the main driver of the associations observed. The results were also consistent when including the three characteristics associated with switching (post-hoc analysis), namely age (OR 0.93, 95% CI: 0.86–1.00 per year, *p* = 0.054), blood eosinophils (OR 0.86, 95% CI: 0.73–1.00 per µL^−1^, *p* = 0.054), and OCS dose (OR 1.11, 95% CI: 1.03–1.20 per mg of daily prednisone, *p* = 0.006) (n. of subjects in the model = 57).

**Figure 1:**
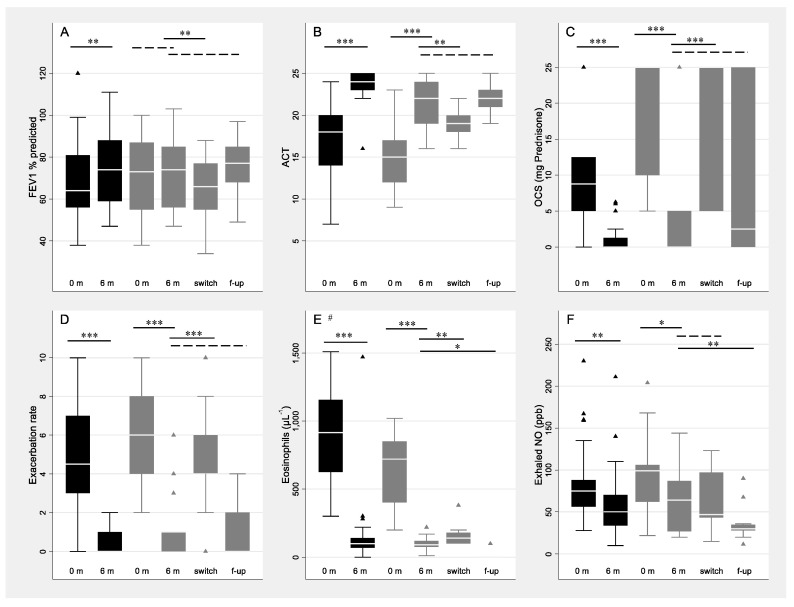
Distribution of clinical characteristics of patients ((**A**): lung function expressed as FEV1%; (**B**): ACT—Asthma Control Test; (**C**): Oral Corticosteroid daily intake, expressed as mg of prednisone; (**D**): asthma exacerbation rate; (**E**): blood eosinophils count; (**F**): Fractional Exhaled Nitric Oxide) at baseline and follow-up examinations by subgroup: non-switch (black boxes) and switch (grey boxes). 0 m, baseline examination; 6 m, examination at 6 months; switch, examination at the time of switching; f-up, final follow-up examination; triangles represent outliers; * *p* < 0.05, ** *p* < 0.01, *** *p* < 0.001 for comparisons with the examination at 6 months; dashed lines indicate *p* > 0.05. #, one point omitted for graphical reasons (eosinophils = 15,000 per µL for one patient of the non-switch subgroup, baseline examination).

All the patients showed an optimal response to mepolizumab according to all the evaluated clinical variables (Figure 1), up to six months. In particular, when considering the parameters representing criteria for switching as stated above, a significant reduction of OCS daily intake between baseline and follow-up was observed in both the non-switch (median [Q1–Q3]: from 8.8 [5.0–12.5] mg to 0.0 [0.0–1.3] mg, *p* < 0.001) and the switch subgroups (from 25.0 mg [10.0–25.0] mg to 0.0 [0.0–5.0] mg, *p* < 0.001). A significant reduction was also observed in the exacerbation rate in both the non-switch (from 4.5 [3.0–7.0] to 0.0 [0.0–1.0], *p* < 0.001) and the switch subgroups (from 6.0 [4.0–8.0] to 1.0 [0.0–1.0], *p* < 0.001).

The need for switching, according to the above-mentioned criterion, occurred for 30 patients after a median time of 21 months (Q1–Q3: 12–24) from mepolizumab initiation. At the time of switching, a significant worsening of most the outcomes was detected in comparison to the 6 months assessment (Figure 1). At the follow-up time-point after the switch (median time: 31 months (Q1–Q3: 22–35), all the outcomes substantially improved and no cases of poor clinical response to benralizumab were detected. In particular, blood eosinophil concentrations and Exhaled NO levels were even lower than the 6-months evaluation (Figure 1E,F).

## 4. Discussion

Little real-life evidence focused on the clinical profile of responders to benralizumab after ineffective mepolizumab treatment. A large study described benralizumab efficacy in patients not responding to mepolizumab or reslizumab, without any subgroup-focused analysis, thus not excluding the potential confounding effect related to the weight-adjusted reslizumab dose [4]. In another report, the coexistence of eosinophilic chronic rhinosinusitis in mepolizumab responders was associated with an even higher response when switching to benralizumab [5]. A case series retrospectively investigated patients experiencing poor response to mepolizumab and successfully managed with benralizumab; however, timing of switching and potential baseline predictors of better response to benralizumab were not addressed [6].

The proportion of late non-responders to mepolizumab we have described in our report seems not to be in line with other studies on the long-term efficacy of the same drug [8,9]. COLUMBA was a multicentre open-label long-term safety study including 362 patients previously enrolled in the DREAM randomized double-blind placebo-controlled trial [8]. Over the COLUMBA study, the mean duration of treatment was 3.5 years. According to the main findings, a sustained safety and efficacy of mepolizumab could be demonstrated. The poor comparability between randomized clinical trials and real life studies in terms of study population has been highlighted [10,11], providing a major explanation for the discrepancy with our findings. In addition COLUMBA study was designed for safety outcomes assessment, which reduces the strength of efficacy outcomes results. However, a gradual decline of FEV1 overtime is reported by the authors, suggesting a potential reduction of mepolizumab efficacy on lung function in some patients, which is in line with our data. Furthermore, no data about OCS daily intake are available from the study, which hamper a direct comparison with our observations.

REALITI-A is a prospective single arm observational cohort study aiming to collect real-world data on asthma patients undergoing mepolizumab treatment. The initial analysis [9] on 368 subjects demonstrated a sustained effectiveness of mepolizumab over the follow-up period in terms of exacerbation rate and daily OCS dose. Of note, when comparing the baseline characteristics of REALITI-A patients and our switch subgroup, the last seems to be affected by a more severe disease in terms of OCS daily dose (median [IQR]: 10.0 (5.0–15.0) vs. 25 (12.5–25)). Furthermore, the available data on REALITI-A refers to a 12-months follow-up observation, while in our study the need for switching occurred after a median time of 21 months. In addition, 20% of REALITI-A population had been treated with omalizumab previous to mepolizumab initiation, actually representing a “switch subgroup”.

In fact, in our study, the timing of poor response onset suggests that in a subset of patients initially well controlled by mepolizumab a change in the immunological background occurred. According to the low levels of blood eosinophils even at the switch time-point the development of anti-drug antibodies is unlikely. The known heterogeneity of inflammatory phenotype, which is intra-individually dynamic over time in asthma patients, may account for the “late non-response” [12]. Under that perspective, an IL-5 independent eosinophilic inflammation can be hypothesized. In fact, eosinophils express on their surface a number of receptors for different cytokines, including epithelial cytokines, IL-3 and GS-CMF, which also play a pivotal role in T2 inflammation and in triggering eosinophils. Furthermore, some evidence supports the presence of IL-5 receptor on ILC-2 and basophils, major cellular players in severe asthma [13].

What mentioned above may account for the different clinical response following the inhibition of IL-5 or the interaction with all the cells expressing its receptor, besides eosinophils, and substantially contributing to the T2 high inflammation.

On clinical grounds, a higher OCS use has been described by Bleecker et al. as a predictor of better clinical response to benralizumab, independently of baseline blood eosinophilia, in the pooled-data analysis from the SIROCCO and CALIMA benralizumab trials [14]. OCS use was associated with a significantly higher probability of switching in our population. Why a higher eosinophilic blood count was related with a lower risk of switching in our study (OR 0.85, 95% CI: 0.73–0.99) is difficult to explain. In this regard the daily OCS dose, much higher in the “switch” subpopulation, may represent a confounding factor. When correcting the analysis for OCS dose (Table 1), the association between eosinophilic blood count and switching was no longer statistically significant, but the estimated association only slightly shifted to null (OR 0.88, 95% CI: 0.75–1.05), suggesting that the blood eosinophil count may have an independent effect on the occurrence of switching. In fact, a poor correlation between blood count and eosinophilic tissue infiltration has been highlighted [14], so that a significant tissue eosinophilia can be hypothesized to exist in patients more at risk of switching. Furthermore, the above-mentioned complex cellular and humoral cross-talk in T2 high severe asthma besides eosinophils may account for our observation.

The small sample size and the retrospective design represent major limitations. Further studies with larger sample sizes and prospective design are needed to confirm our findings and to reinforce their external validity and applicability to other populations.

However, to our knowledge, our study provides the first real-word focus on clinical variables potentially predicting a better response to anti IL-5r in patients fully eligible for both mepolizumab and benralizumab.

## 5. Conclusions

Taken together, our data suggest that in patients eligible to monoclonal antibodies interfering with Il-5 driven cascade, younger age, higher OCS daily dose and lower blood eosinophils at baseline were associated with a significantly higher risk for switching from mepolizumab to benralizumab, somehow predicting a better response to anti IL-5r. Furthermore, according to our observations, in late non-responder patients to mepolizumab, more robustly targeting the IL-5 axis may be effective.

Overlapping eligibility and monoclonal antibody switching deserves larger investigations, also by exploring data from the national and international severe asthma registries, and consensus recommendations due to its major clinical and pharmaco-economical relevance.

## Figures and Tables

**Table 1:** Baseline characteristics of study population subgroups (non-switch/switch) and their association with the occurrence of switching ^a^.

Baseline Characteristic	Missing Data (*n*, %)	Non-Switch Subgroup (*n* = 38)	Switch Subgroup (*n* = 30)	Unadjusted OR (95% CI)	OR (95% CI) Adjusted for Daily OCS Dose at Baseline
Age (Years)	-	58 (53, 67)	52.5 (45, 57)	0.93 (0.89–0.98) **	0.95 (0.89–1.01)
Sex (Female, n) (%)	-	22 (58)	21 (70)	1.70 (0.62–4.67)	1.58 (0.46–5.46)
BMI, kg/m^2^	4 (6%)	24.1 (22.1, 26.7)	22.8 (20.8, 27.0)	0.99 (0.86–1.13)	1.03 (0.87–1.22)
Nasal polyps (*n*, %)	-	17 (45)	19 (63)	2.13 (0.80–5.68)	2.54 (0.76–8.55)
FEV_1_, L	2 (3%)	1.9 (1.3, 2.4)	1.9 (1.3, 2.2)	0.97 (0.46–2.02)	0.77 (0.31–1.93)
FEV_1_ % predicted	3 (4%)	64 (56, 81)	63.5 (48, 77)	0.99 (0.96–1.02)	0.98 (0.95–1.02)
Tiffeneau Index, %	2 (3%)	64 (59, 68)	63 (54, 75)	1.01 (0.97–1.06)	1.00 (0.94–1.05)
FeNO, ppb	5 (7%)	74.5 (54, 88)	62 (35, 102)	0.97 (0.88–1.08) ^b^	0.97 (0.86–1.10) ^b^
Blood eosinophils, µL^−1^	5 (7%)	860 (560, 1140)	490 (340, 750)	0.85 (0.73–0.99) ^c^ *	0.88 (0.75–1.05) ^c^
ACT score	1 (1%)	18 (14, 20)	14.5 (12, 18)	0.92 (0.83–1.03)	0.96 (0.85–1.08)
Exacerbations in the last year (n)	-	4.5 (3, 7)	5 (4, 8)	1.07 (0.90–1.28)	0.96 (0.78–1.19)
OCS, daily prednisone dose (mg)	6 (9%)	7.5 (5, 12.5)	25 (12.5, 25)	1.15 (1.07–1.24) ***	-

ACT: Asthma Control Test; BMI: Body max Index; FEV_1_: Forced Expiratory Volume in 1 second; FeNO: Fractional exhaled Nitric Oxide; OCS: Oral Corticosteroid; OR: Odds ratio; ^a^ for quantitative variables median values (Q1, Q3) are reported; ^b^ OR for 10 ppb increase; ^c^ OR for 100 units/µL increase; * *p* < 0.05, ** *p* < 0.01, *** *p* < 0.001.

## Data Availability

Data are available upon motivated request to the corresponding author.
